# Transgenerational and intergenerational effects of early childhood famine exposure in the cohort of offspring of Leningrad Siege survivors

**DOI:** 10.1038/s41598-023-37119-8

**Published:** 2023-07-11

**Authors:** Kristina Tolkunova, Dmitrii Usoltsev, Ekaterina Moguchaia, Maria Boyarinova, Ekaterina Kolesova, Anastasia Erina, Trudy Voortman, Elena Vasilyeva, Anna Kostareva, Evgeny Shlyakhto, Alexandra Konradi, Oxana Rotar, Mykyta Artomov

**Affiliations:** 1 Almazov National Medical Research Centre, Saint Petersburg, Russia; 2 ITMO University, St. Petersburg, Russia; 3 Department of Epidemiology, Erasmus MC, University Medical Center, Rotterdam, The Netherlands; 4 Institute for Genomic Medicine, Nationwide Children’s Hospital, Columbus, OH USA; 5 Department of Pediatrics, College of Medicine, Ohio State University, Columbus, OH USA

**Keywords:** Nutrition, Epidemiology

## Abstract

Famine exposure during early life development can affect disease risk in late-life period, yet, transmission of phenotypic features from famine-exposed individuals to the next generations has not been well characterized. The purpose of our case–control study was to investigate the association of parental starvation in the perinatal period and the period of early childhood with the phenotypic features observed in two generations of descendants of Leningrad siege survivors. We examined 54 children and 30 grandchildren of 58 besieged Leningrad residents who suffered from starvation in early childhood and prenatal age during the Second World War. Controls from the population-based national epidemiological ESSE-RF study (n = 175) were matched on sex, age and body mass index (BMI). Phenotypes of controls and descendants (both generations, children and grandchildren separately) were compared, taking into account multiple testing. Comparison of two generations descendants with corresponding control groups revealed significantly higher creatinine and lower glomerular filtration rate (GFR), both in meta-analysis and in independent analyses. The mean values of GFR for all groups were within the normal range (GFR less than 60 mL/min/1.73 m^2^ was recorded in 2 controls and no one in DLSS). Additionally, independent of the creatinine level, differences in the eating pattern were detected: insufficient fish and excessive red meat consumption were significantly more frequent in the children of the Leningrad siege survivors compared with controls. Blood pressure, blood lipids and glucose did not differ between the groups. Parental famine exposure in early childhood may contribute to a decrease in kidney filtration capacity and altered eating pattern in the offspring of famine-exposed individuals.

## Introduction

The theory of fetal origin of health and diseases suggests that parental environmental exposure during fetal development may be an important risk factor for adult-onset diseases, in particular, for cardiovascular diseases (CVD)^1^. Susceptibility to diseases resulting from fetal programming can be observed directly after birth in exposed individuals or transmitted from the exposed generation to the descendants^2^.

Major famine studies of the Dutch Famine 1944–1945 and the Chinese Famine 1959–1962 have shown that insufficient nutrition during prenatal and early life periods have critical impacts on fetal programming. Importantly, not only were individuals directly exposed to famine during the prenatal period shown to have phenotypic consequences of exposure in later life. It was suggested that the effect of fetal programming can be transmitted to the descendants of exposed individuals, potentially through epigenetic mechanisms^3^. For example, the Dutch Hunger study observed a higher frequency of neonatal obesity in children of women exposed to malnutrition prenatally and a higher body mass index (BMI) at an older age in the offspring of prenatally malnourished men^4,5^. The China Health and Nutrition Survey reported significantly lower glomerular filtration rate (GFR), and a faster than expected increase in BMI with age, waist circumference, and blood pressure (BP) in the first generation of descendants of exposed individuals^6,7^.

The Siege of Leningrad was another tragic example of catastrophic malnutrition of the city's residents having experienced a major supply deficit for two and a half years during the Second World War^8^. There are some similarities but also difference with the two other major famine studies: The Siege of Leningrad was longer than Dutch Famine (28 months compared to about 6 months, respectively), the weather was significantly colder, affecting the calorie demand and potential hypothermia, and daily rations were not restored after breaking the blockade (in the Netherlands daily rations quickly rose within a week)^9^. The Chinese famine, which is comparable in duration to the famine during the Siege of Leningrad, was characterized by pandemic nature and uneven intensity distribution within the country^10^.

The purpose of our study was to investigate the association of starvation in the prenatal period and the period of early childhood with phenotypic differences later in life in two generations of descendants of Leningrad Siege survivors (DLSS).

## Materials and methods

**Figure 1:**
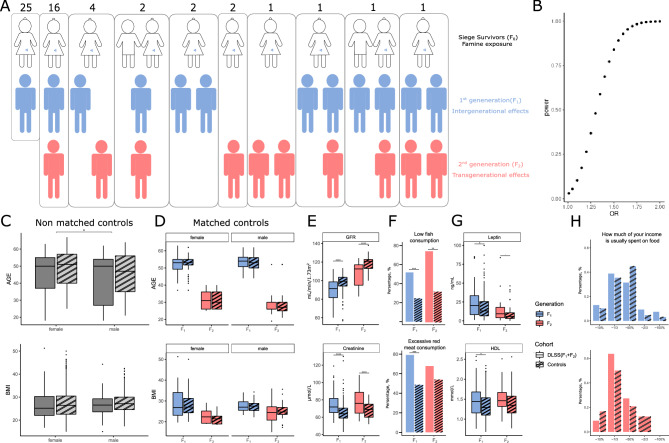
Comparison of descendants of Leningrad Siege survivors (DLSS) with population controls from St. Petersburg. (**A**) Graphical representation of all family trees in the DLSS cohort (Siege survivors—F_0_, the 1st generation of descendants—F_1_, the 2nd generation of descendants—F_2_). (**B**) Power calculations. (**C**) Non-matched controls and DLSS. (**D**) Matched controls and DLSS. (**E**) Differences in GFR and creatinine levels (**F**) red meat and fish consumption (**G**) leptin and HDL levels between each generation of DLSS and the corresponding controls from the populational cohort of St. Petersburg. (**H**) Differences in income between each generation of DLSS and the corresponding controls from the populational cohort of St. Petersburg.

In 2009–2011, 305 Leningrad siege survivors who starved in early childhood and/or in utero during the Second World War (1941–1944) were examined in the Almazov National Medical Research Centre as part of an observational cohort study, the design and general characteristics were described in previous publications^8^*.* In 2020–2021, we examined 87 adult DLSS (57 children and 30 grandchildren from 55 families) of 58 Siege survivors, with the current pregnancy being the only exclusion criteria (Fig. 1A). A subsample of a population-based cohort (N = 1600) of St. Petersburg residents aged 25–64 years within the national epidemiological study ESSE-RF^11^ were selected as controls by matching gender, age and BMI distribution to each generation of descendants*.*

Conventionally, if a man or a woman (F0) and their germ cells that will form the F1 generation are directly exposed to an environmental stressor this constitutes the intergenerational effect. The F2 offspring is the first generation that is not directly exposed to the stressor and represents transgenerational epigenetic effect^12^. In the current study we refer to Leningrad Siege survivors exposed to starvation in early life as generation zero (F_0_), their children as the first generation of DLSS (F_1_) and grandchildren as the second generation (F_2_). The final control cohorts included 127 individuals for F_1_ and 48 for F_2_.

The study was approved by the Local Ethics Committee of the Almazov National Medical Research Centre (Protocol #2401-21 from the local ethics committee meeting #01-21 dated January 18, 2021). All the participants have signed an informed consent. Research involving human research participants must have been performed in accordance with the Declaration of Helsinki.

Body weight measurement was carried out on the scales of the brand VEM-150-Mass-K (Russia), height—using the height meter RM-1 Diacoms (Russia), waist and hip circumference—using a standard flexible centimeter tape. To define respondents as obese or non-obese we use two relevant indicators: waist circumference and BMI. Abdominal obesity was determined according to the JIS 2009 metabolic syndrome criteria 2009^13^ as: waist circumference ≥ 94 cm for males and ≥ 80 cm for females. The BMI was calculated as the ratio of body weight in kilograms to height in meters squared. All respondents were classified as obese (BMI ≥ 30 kg/m^2^) or non-obese (BMI < 30 kg/m^2^).

Double measurement of blood pressure (BP) on the right arm was performed with the automatic tonometer “Omron” (Japan) with intervals of 2 min, after resting for 5 min in a sitting position, then a single measurement of BP was made 3 min after verticalization was made. Hypertension was diagnosed if the mean BP level was ≥ 140/90 mmHg and/or if patient was receiving antihypertensive therapy.

The creatinine, blood glucose, lipids study (total cholesterol—TC, low-density lipoproteins—LDL, high-density lipoproteins—HDL, triglycerides—TG) measurement was performed in fasting state (AbbotArchitect 8000 (USA)). The levels of leptin (Leptin ELISA, DBC (Canada)) and adiponectin (Adiponectin ELISA, Mediagnost (Germany)) levels were determined by enzyme immunoassay. We assessed glucose metabolism disorders: participants were divided into fasting hyperglycemia group (glucose level ≥ 5.6 mmol/L) and diabetes mellitus group (the patients self-reported about diabetes). The glomerular filtration rate (GFR) was calculated using the CKD-EPI formula^14^*.* Groups of participants with high total cholesterol (> 4.9 mmol/L), LDL (> 3.0 mmol/L), triglycerides (≥ 1.7 mmol/L), and reduced HDL (in male < 1.0 and in female < 1.2 mmol/L) were also formed; hypolipidemic therapy was taken into account^15^*.*

Behavioral and socioeconomic risk factors were evaluated with questionnaires and interviews: diet pattern, smoking status, alcohol consumption, duration of sleep, level of education, physical activity. The percentage of income spent on food was also taken into account. The consumption of fresh vegetables and fruits was assessed (respondents were divided into those who had fresh fruits and vegetables in their daily diet and those who consumed irregularly). We considered that excess salt intake was the result of salting cooked dishes. Consumption of fish (200 g) less than 1–2 times a week was considered insufficient. Daily consumption of red meat (150 g or more) was considered excessive^16^. If the respondent consumed ≥ 6 pieces/spoons of sugar per day or daily/almost daily intake of sweets/confectionery, it was considered excessive intake of sweets. The duration of sleep of less than 6 h a day was considered insufficient. Moderate physical activity ≥ 150 min per week was sufficient. Inactivity was recorded in the case of being in a sitting position for more than 9 h on a weekday. The assessment of smoking status was also carried out, respondents were divided into groups: smoking at the moment, in the past, and those who have never smoked. The number of alcohol doses and the frequency of alcohol consumption were assessed and respondents were divided into those who regularly took alcoholic beverages and those who did not consume at all.

Mathematical and statistical data analysis was implemented using the R-4.0 programming language^17^ and libraries dplyr (v1.0.7)^18^, ggplot2 (v3.3.5)^19^, tidyr (v1.1.4)^20^. R library powerMediation (v0.3.4)^21^ was used to compute the power of the logistic model for detecting odds ratio (OR) in a range from 1.01 to 2. Quantitative parameters were described using median values and lower and upper quartiles (Q1–Q3). Nominal data were described with absolute values and percentages.

The prevalence of 44 phenotypic risk factors in both generations of DLSS was simultaneously assessed using a logistic model adjusted for sex, generation and BMI. In case of absence of a phenotype in one of the comparison groups, a simple Fisher test was used instead of logistic regression. We report both effect size (beta), and odds ratio for each phenotype. For statistically significant phenotypes we also reported beta standard error (se). If a phenotype was obtained from the other phenotype through calculations, we considered the pair as a single phenotype for multiple hypothesis correction with Bonferroni approach. In total, 34 independent phenotypes were analyzed and the significance threshold for the p-value was 0.05/34 = 0.0015.

## Results

87 DLSS (mean age: 43.8 years; sd: ± 13.4 years; range: 18–63 years; 44% men) were observed during an ambulatory visit at Almazov National Medical Research Centre (St. Petersburg, Russia). The prevalence of abdominal obesity did not differ significantly among BMI-matched participants. The BP levels and the prevalence of hypertension were insignificant compared to population average. The DLSS group had nominally higher HDL levels. The differences are described in more details in the supplement and Sup. Tab. S1. Our power calculation showed that we have 80% power to detect signals as small as OR = 1.47 (Fig. 1B).

The DLSS cohort had 57 and 30 individuals from F_1_ and F_2_, respectively (Fig. 1A). Initially we performed matching of the DLSS cohort to a group of 1600 controls from the ESSE population cohort from St. Petersburg. We excluded individuals with known cardiovascular diseases and outliers (more than 6 standard deviations) based on blood biochemical analyses and blood pressure measurements from the control pool. The final control pool available for matching had 1142 individuals (Fig. 1C).

Controls of the same gender from the ESSE populational cohort were selected for each individual so that the age and BMI of the controls did not differ for more than two units. No controls were found for 8 DLSS individuals (7 youngest individuals from F_2_) and (1 individual with high BMI from F_2_), therefore they were excluded from further analyses. 3 individuals from F_1_ of DLSS were excluded because they had cardiovascular disease. The final cohort included a total of 175 controls and 76 DLSS (127 controls and 54 DLSS for F_1_; 48 controls and 22 DLSS for F_2_, Fig. 1D).

Of all the biomarker phenotypes that were analyzed and compared, only creatinine (p = 1.367 × 10^−7^, beta = 0.092, se = 0.017) and GFR (p = 3.94 × 10^−8^, beta = − 0.08, se = 0.015) passed the Bonferroni significance threshold (Fig. 1E, Sup. Tab. S1). GFR less than 60 mL/min/1.73 m^2^ was recorded in 2 controls and no one in DLSS. Additionally, insufficient fish consumption was more frequently observed in the DLSS group compared to controls (p = 3.15 × 10^−6^; beta = 1.361; beta se = 0.292), and also excessive red meat consumption was more frequently observed in the DLSS group (p = 1.8 × 10^−4^; beta = 1.168; beta se = 0.312) (Fig. 1F, Sup. Tab. S1). Among cardiometabolic risk factors, nominal differences were observed for leptin and HDL levels that were higher in the DLSS group, but still within normal ranges (leptin: p = 3.12 × 10^−3^, beta = 0.037; HDL: p = 9.47 × 10^−3^, beta = 1.08) and not significant after multiple hypothesis testing correction (Fig. 1G, Sup. Tab. S1). Also, we found nominally smaller frequency of excessive salt consumption among the DLSS group (p = 0.0056, beta = − 0.814) and a higher frequency of diabetes mellitus (P = 6.82 × 10^−3^, beta = 2.33). There were also no differences in the prevalence of hypertension, and abdominal obesity. Additionally, we detected that neither blood pressure (P = 0.73) nor diabetes (P = 0.55) were predictors for GFR.

Further, we investigated whether this effect could be observed in each generation separately. We analyzed the phenotypic data within each generation independently, adjusting the model for age, BMI, sex. Interestingly, the effect of creatinine and GFR was observed in both generations—F_1_: creatinine (p = 9.71 × 10^−5^, beta = 0.071, se = 0.018) and GFR (p = 1.09 × 10^−5^, beta = − 0.076, se = 0.017) and F_2_: creatinine (p = 3.56 × 10^−4^, beta = 0.208, se = 0.058) and GFR (p = 4.56 × 10^−4^, beta = − 0.179, se = 0.051) (Sup. Tab. S1). The differences in fish and red meat intake habits passed Bonferroni correction only for F_1_ (red meat: p = 3.10 × 10^−4^; beta = 1.39; beta se = 0.385. Fish: p = 5.23 × 10^−4^; beta = 1.197; beta se = 0.345), but estimates for F2 were in similar direction and magnitude (red meat: p = 0.237; beta = 0.653; beta se = 0.55. Fish: p = 0.0015; beta = 1.897; beta se = 0.598) (Sup. Tab. S1). Additionally nominal longer mean sleep duration was registered only in F2 (p = 0.0318, beta = 0.483). We didn’t observe any differences between DLSS and controls in income (Fig. 1H).

**Figure 2:**
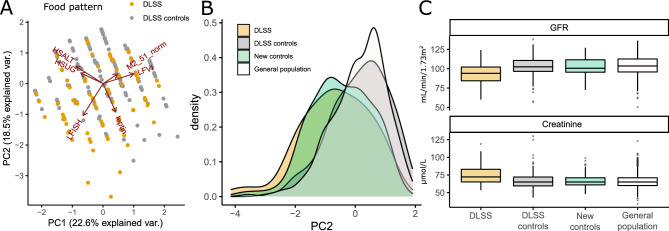
Food consumption pattern. (**A**) The first two principal components (PC) of food consumption pattern and loadings. (**B**) The distribution of PC2 in different cohorts. (**C**) The distribution of GFR and creatinine levels in different cohorts.

In addition to the consumption of red meat and fish, several other nutritional phenotypes were nominally significant, therefore, we used the principal component analysis (PCA) based on food consumption-related phenotypes (fish, red meat, salt, sugar, vegetable intake, and leptin) to investigate differences in food pattern between DLSS and controls (Fig. 2A). PC2 was characterized by a low fish intake, leptin levels, and high salt and sugar intake. It was significantly different in descendants and controls for both the meta-analysis (p = 3.77 × 10^−9^; beta = − 1.06; beta se = 0.18) and for each generation separately: F_1_ (p = 2.15 × 10^−6^; beta = − 1.00; beta se = 0.211); F_2_ (p = 5.29 × 10^−4^; beta = − 1.243; beta se = 0.359) (Sup. Tab. S1, S2).

Subsequently, we investigated whether the food pattern was related to the observed difference in creatinine level. From the control population that was not used in the previous comparisons, we selected individuals to match sex, age, and PC2 distributions obtained from the PCA of food intake in the DLSS cohort (Fig. 2B). Comparison of this cohort with the previously selected control cohort for DLSS comparison, we did not observe significant differences in creatinine and GFR (p = 0.75; p = 0.61, respectively, Fig. 2C). Therefore, the pattern of food consumption and biochemical markers of renal function can be interpreted as independent associations.

## Discussion

Our results demonstrate a significantly higher creatinine level and a lower GFR in the descendant generations of individuals exposed to prenatal or early childhood starvation. It is known that most human organs have a critical development period at the prenatal stage. In the case of serious nutritional deficiency, the number of functioning glomeruli decreases, leading to increased filtration load, increased GFR throughout postnatal life, and subsequent damage to functioning glomeruli^22^. Reports on the Northern China famine cohort study also presented significantly lower calculated GFR in prenatally exposed participants to starvation and their children compared to controls. Data on the second generation were not available in this study. It has been hypothesized that the starvation of the ancestors in early childhood contributes to a decrease in the renal filtration capacity in their offspring. This may be due to transgenerational changes in the epigenome through the programming of nephron deficiency in germ cells^6^. In the long term, renal filtration capacity can cause glomerulosclerosis, and finally hypertension in adulthood^22^. It is important to note that, according to our data, DM and hypertension were not the predictors of a decrease in GFR.

Observed differences in the dietary pattern between the descendants and controls do not have a clear direction of effect. On the one hand, increased red meat and decreased fish consumption increase the risk of cardiometabolic disorders^23,24^. On the other hand, the decreased salt consumption might indicate a healthier approach to food selection. It is important to note that there were no statistically significant differences in terms of income share spent on food. Comparison of the subsample of the population cohort that matched in dietary behavior with DLSS with the control population indicated that the observed differences in creatine levels and food selection were independent of each other. Due to the fact that all the respondents in the cohort are from the same metropolitan area, they are exposed to quite homogeneous environmental factors and have relatively similar diet and access to healthcare. Based on the aforesaid, the geographical location is unlikely to contribute to the results, although it has to be interpreted with caution.

It is known that kidney function and GFR as its indicator depend on the diet. For example, diets rich in saturated fat are associated with a decrease in the number and size of glomeruli, as well as a large decrease in GFR and, on opposite, consumption of fruit, vegetables, and dietary fiber (DASH and Mediterranean diets) showed associations with a low risk of developing chronic kidney disease^25^. Within the control population we selected the group of individuals with a similar dietary pattern to DLSS and observed no notable differences in kidney function, therefore, confirming that the difference in kidney function between DLSS and controls could not be attributed to dietary behavior.

The absence of statistically significant differences in the prevalence of cardiometabolic diseases can be explained on the one hand by the small size of the cohort studied, and on the other hand by the relatively young age of most of the participants. Also, it is important to note that in this work we studied the offspring, among whom most of the parents are still alive, which may have resulted in survivorship bias. In addition, both a decrease in fertility and an increase in perinatal mortality during famine may have led to selective births of healthier or more resilient individuals. The besieged Leningrad residents included in the study could therefore represent an artificially "healthy" sample.

The prenatal effects of starvation have also been studied in other cohorts. Study of the Chinese famine showed the early exposure to famine was associated with increase in BMI, waist circumference, and BP with sex and age differences. The effects of experienced starvation were stronger in men compared with females and older subjects compared with younger ones^7^. The study also revealed a significant increase of hyperglycemia risk in 2 consecutive generations of Chinese adults as a result of prenatal exposure to famine^26^. In the Dutch cohort study on the effects of perinatal starvation, the authors found no transgenerational effects of prenatal exposure to malnutrition on birth weight, or on the incidence of cardiovascular and metabolic diseases. But the early exposure to starvation was associated with an increase in neonatal obesity of the second generation that might be the evidence of increased obesity and diabetes risk in further life^4^. Another study found no difference in the incidence of cardiovascular disease, hypercholesterolemia, diabetes mellitus, and hypertension between male and female offspring. But prenatal exposure to starvation in men was associated with higher body mass index in their offspring^5^. However, the authors did not distinguish between intergenerational and transgenerational effects in the offspring cohort. We examined two generations of offspring from parents who were exposed to starvation. The first generation elucidates intergenerational effects of starvation. The second generation was not exposed directly to the effects of starvation, even at the stage of development of parental female reproductive cells representing transgenerational effects of starvation^5^.

Conclusively, observation of phenotypic effects associated with early-in-life exposures, separated not only in time, but also by generations serve yet another example of Barker’s hypothesis of fetal origins of chronic diseases of adult life. Our study suggests that not only individuals directly exposed to traumatic events, but their offspring will benefit from additional health screenings, in particular assessment of kidney function, for earlier detection of disease symptoms and their successful treatment.

## Supplementary Information

Supplementary Tables.

Supplementary Information.

## Data Availability

Raw clinical data is not permitted for sharing according to the terms of the informed consent. The summary information or other non-subject level analyses for the datasets used and/or analyzed during the current study available from the corresponding author on reasonable request.

## Abbreviation

DLSS: Descendants of Leningrad Siege survivors

## References

[1] Barker DJ (1995). Fetal origins of coronary heart disease. BMJ.

[2] Cavalli G, Heard E (2019). Advances in epigenetics link genetics to the environment and disease. Nature.

[3] Öztürk HNO, Türker PF (2021). Fetal programming: Could intrauterine life affect health status in adulthood?. Obstet. Gynecol. Sci..

[4] Painter RC, Osmond C, Gluckman P, Hanson M, Phillips DI, Roseboom TJ (2008). Transgenerational effects of prenatal exposure to the Dutch famine on neonatal adiposity and health in later life. BJOG.

[5] Veenendaal MVE, Painter RC, de Rooij SR, Bossuyt PM, van der Post JA, Gluckman PD (2013). Transgenerational effects of prenatal exposure to the 1944–45 Dutch famine. BJOG.

[6] Jiang W, Han T, Duan W, Dong Q, Hou W, Wu H (2020). Prenatal famine exposure and estimated glomerular filtration rate across consecutive generations: Association and epigenetic mediation in a population-based cohort study in Suihua China. Aging (Albany NY)..

[7] Li J, Yang Q, An R, Sesso HD, Zhong VW, Chan KHK (2022). Famine and trajectories of body mass index, waist circumference, and blood pressure in two generations: Results from the CHNS from 1993–2015. Hypertension.

[8] Rotar O, Moguchaia E, Boyarinova M, Kolesova E, Khromova N, Freylikhman O (2015). Seventy years after the siege of Leningrad: Does early life famine still affect cardiovascular risk and aging?. J. Hypertens..

[9] Fredrick J, Stare MD (1945). Nutritional conditions in Holland. Nutr. Rev..

[10] Susser E, St CD (2013). Prenatal famine and adult mental illness: Interpreting concordant and discordant results from the Dutch and Chinese Famines. Soc. Sci. Med..

[11] Orlov AV, Rotar OP, Boyarinova MA, Alieva AS, Dudorova EA, Kolesova EP (2015). Gender characteristics of the prevalence of behavioral risk factors in St. Petersburg residents. Bull. Russ. Acad. Med. Sci..

[12] Skinner MK (2008). What is an epigenetic transgenerational phenotype? F3 or F2. Reprod. Toxicol..

[13] Alberti KG, Eckel RH, Grundy SM, Zimmet PZ, Cleeman JI, Donato KA, International Diabetes Federation Task Force on Epidemiology and Prevention; National Heart, Lung, and Blood Institute; American Heart Association; World Heart Federation; International Atherosclerosis Society; International Association for the Study of Obesity (2009). Harmonizing the metabolic syndrome: A joint interim statement of the International Diabetes Federation Task Force on Epidemiology and Prevention; National Heart, Lung, and Blood Institute; American Heart Association; World Heart Federation; International Atherosclerosis Society; and International Association for the Study of Obesity. Circulation.

[14] Moiseev VC, Mukhin NA, Smirnov AV, Kobalava JD, Bobkova IN, Villevalde SV (2014). Cardiovascular risk and chronic kidney disease: Cardio-nephroprotection strategies. Russ. J. Cardiol..

[15] Kobalava ZD, Konradi AO, Nedogoda SV, Shlyakhto EV, Arutyunov GP, Baranova EI (2020). Arterial hypertension in adults Clinical guidelines 2020. Russ. J. Cardiol..

[16] Visseren FLJ, Mach F, Smulders YM, Carballo D, Koskinas KC, Bäck M, ESC National Cardiac Societies; ESC Scientific Document Group (2021). 2021 ESC Guidelines on cardiovascular disease prevention in clinical practice. Eur. Heart J..

[17] R Core Team. R: A language and environment for statistical computing. https://www.R-project.org/ (R Foundation for Statistical Computing, 2021).

[18] Wickham, H., François, R., Henry, L. & Müller, K. dplyr: A grammar of data manipulation. R package version 1.0.7. https://CRAN.R-project.org/package=dplyr (2021).

[19] Wickham, H. *ggplot2: Elegant Graphics for Data Analysis* (Springer, 2016). ISBN: 978-3-319-24277-4.

[20] Wickham, H. & Henry, L. tidyr: Tidy Messy Data. R package version 1.1.4. https://CRAN.R-project.org/package=tidyr (2021).

[21] Qiu, W. powerMediation: Power/Sample Size Calculation for Mediation Analysis. R package version 0.3.4. https://CRAN.R-project.org/package=powerMediation (2021).

[22] Lejarraga H (2019). Perinatal origin of adult diseases. Arch. Argent. Pediatr..

[23] Bechthold A, Boeing H, Schwedhelm C, Hoffmann G, Knüppel S, Iqbal K (2019). Food groups and risk of coronary heart disease, stroke and heart failure: A systematic review and dose-response meta-analysis of prospective studies. Crit. Rev. Food Sci. Nutr..

[24] Pan L, Chen L, Lv J, Pang Y, Guo Y, Pei P (2022). Association of red meat consumption, metabolic markers, and risk of cardiovascular diseases. Front. Nutr..

[25] Quintela BCSF, Carioca AAF, de Oliveira JGR, Fraser SDS, da Silva Junior GB (2021). Dietary patterns and chronic kidney disease outcomes: A systematic review. Nephrology (Carlton).

[26] Li J, Liu S, Li S, Feng R, Na L, Chu X, Wu X, Niu Y, Sun Z, Han T, Deng H, Meng X, Xu H, Zhang Z, Qu Q, Zhang Q, Li Y, Sun C (2017). Prenatal exposure to famine and the development of hyperglycemia and type 2 diabetes in adulthood across consecutive generations: A population-based cohort study of families in Suihua, China. Am. J. Clin. Nutr..

