# Supplementary material for: Transgenerational and intergenerational effects of early childhood famine exposure in the cohort of offspring of Leningrad Siege survivors
Source: Sci Rep. 2023 Jul 11;13:11188. doi: 10.1038/s41598-023-37119-8 (PMC10336086; doi:10.1038/s41598-023-37119-8)
Supplement: Supplementary file 2 — Supplementary Information. [file 41598_2023_37119_MOESM2_ESM.docx]

**TRANSGENERATIONAL AND INTERGENERATIONAL EFFECTS OF EARLY CHILDHOOD FAMINE EXPOSURE IN THE COHORT OF OFFSPRING OF LENINGRAD SIEGE SURVIVORS.**

Kristina Tolkunova^1*^, Dmitrii Usoltsev^1,2*^, Ekaterina Moguchaia^1^, Maria Boyarinova^1^, Ekaterina Kolesova^1^, Anastasia Erina^1^, Trudy Voortman^3^, Elena Vasilyeva^1^, Anna Kostareva^1^, Evgeny Shlyakhto^1^, Alexandra Konradi^1,2^, Oxana Rotar^1,3,#^, Mykyta Artomov^1,2,4,5,#^

1 - Almazov National Medical Research Centre, Saint Petersburg, Russia

2 - ITMO University, St. Petersburg, Russia

3 - Erasmus Medical Center, Rotterdam, The Netherlands

4 - Institute for Genomic Medicine, Nationwide Children’s Hospital, Columbus, OH, USA

5 - Department of Pediatrics, College of Medicine, Ohio State University, Columbus, OH, USA

* - contributed equally

^#^ - Correspondence: mykyta.artomov@nationwidechildrens.org, oxana.rotar@gmail.com

**The authors declare no conflict of interest**

**Table of contents**

**Controls Matching and power calculations 3**

**Phenotypic analysis 4**

**Exploration of phenotypic patterns 5**

# **Controls Matching and power calculations.**

We used R to explore 87 descendants of Leningrad Siege survivors (DLSS) cohort and a group of 1,600 control individuals from the ESSE populational cohort ( **Fig. 1C)** (1)**.**

Initially, we excluded all patients with cardio-vascular diseases and patients with more than 6 standard deviations of general biochemical parameters and blood pressure. Only 1,142 individuals were rested as potential controls.

Controls of the same gender from the populational cohort were selected for each individual so that the age and BMI of the controls did not differ more than 2. No controls were found for 8 DLSS individuals (7 young individuals from F_2_) and (1 individual with high BMI from F_2_).3 individuals from F_1_ were excluded because they had cardiovascular disease. Finally, we found 175 controls for 75 cases (127/54 for the first population and 48/22 for the second population) (**Fig. 1D).**

We use R library powerMediation (v0.3.4) (2) to compute the power of the logistic model for detecting OR in a range from 1.01 to 2 (**Fig. 1B)**.

# **Phenotypic analysis**

We used a logistic regression model adjusted for sex generation and BMI to find differences of 44 phenotypic risk factors between DLSS and control groups. If a phenotype was obtained from the other phenotype through calculations, we considered the pair as a single phenotype for multiple hypothesis correction with Bonferroni approach - in total, 34 independent phenotypes were analyzed, therefore significance threshold was defined as 0.05/34=0.0015. Additionally, we implemented these models for each generation independently, replacing the generation correction in model with age.

Only creatinine (p=1.367x10^-7^, beta=0.092, se=0.017), and GFR (p=3.94x10^-8^, beta=-0,08, se=0.015) passed the significance threshold (**Fig. 1E, Sup. Tab. S1**). Additionally, insufficient fish consumption was more frequently observed in the DLSS group compared to controls (p = 3.15x10^-6^; beta = 1.361; beta se=0.292) and excessive red meat consumption were more frequently observed in the DLSS group (p = 1.8x10^-4^; beta=1.168; beta se=0.312) (**Fig. 1F**, **Sup. Tab. S1**)**.**

Also, we detected nominal differences in leptin and HDL levels that were higher in the DLSS group within normal ranges (leptine: p = 3.12x10^-3^; beta=0.037, HDL: p = 9.47x10^-3^; beta=1.08) (**Fig. 1G)**. Also, we found the less excessive salt consumption among DLSS group and more frequent diabetes mellitus (Salt: p=0.0056, beta=-0.814, diabetes: p=0.0068, beta =2.327)

Our previous research demonstrated a higher level of HDL within the normal range in besieged Leningrad residents compared to controls (3). Also, maternal nutrition during pregnancy also plays a key role in the proopiomelanocortin neurons development (satiety key mediator). The proopiomelanocortin neurons of the hypothalamus arched nucleus integrate peripheral signals such as leptin, glucose and insulin, and regulate the energy balance, causing a feeling of satiety and increasing energy consumption (4). According to our results, the level of leptin was higher in Leningrad Siege descendants compared to controls. However, only F1 had higher leptin values compared to the controls, F2 had higher leptin values with the tendency to nominal value. In addition, we registered a higher prevalence of DM in the respondents in the group of DLSS, which was also higher only in the children of exposed parents. The grandchildren of DLSS had no differences in the level of leptin and the prevalence of DM, which may be due to young age. In addition, a longer sleep duration was recorded among the grandchildren of besieged Leningrad residents. It is known that sleep restriction leads to lower leptin levels and increases sympathetic activity and pro-inflammatory cytokines that increase insulin resistance. According to the literature, a U-shaped association of cardio-metabolic outcomes and sleep duration is supposed to exist (5).

Interestingly we didn’t observe any differences between DLSS and controls in income (**Fig. 1H)**

Attitude to low salt intake is one of the demonstrated by DLSS favorable behavioral patterns - they consumed an excessive amount of salt less often with no difference between two generations. According to V.S. Volkov, during the Siege of Leningrad, excessive salt intake was observed and was linked to high mortality due to hypertensive complications (6). Lower salt intake in the Siege of Leningrad survivors might be one of the favorable mechanisms of survival which was passed on to the offspring. Based on our biochemical data, it can be assumed that the descendants of the inhabitants of Besieged Leningrad more often have metabolic disorders, but some favorable behavioral factors may be helpful for preventing the disease's development. Thus, DLSS diet habits can have positive and negative effects on the health of study participants themselves and future generations through epigenetic changes. However, to assess long-term results, a longer follow-up of this cohort is needed.

# **Investigation of phenotypic patterns.**

Since some phenotypes were of nominal significance we combined them into groups (phenotype codes are explained in Sup. Tab. S1): food pattern: HSALT, LFISH, HSUG, LFVI, M2_51_norm, leptin;

biochemical pattern: crea, GFR, adiponectin, chol, Tg, ldlp, hdlp, gluc;

disease pattern: SBP, DBP, AH, M8_6, grCHOL2, grTG2, grLDL2, grHDL2, M8_38, M8_39, AO_94_80, HyperGLU_56new, DIS17;

behavioral pattern: M3_2, SITHIGH, ALC, M4_1a, M4_1b, M4_1c, M7_1, M7_1_dop

For each pattern we independently performed PCA and extracted the first two principal components. Then for each PC we performed logistic regression with the model previously used for phenotypic analysis. We found that PC2 was significantly different (meta-analysis with two generations of descendants: p = 3.77x10^-9^; beta=-1.06; beta se=0.18); (F_1_: p = 2.15x10^-6^; beta=-1.00; beta se=0.211); (F_2_: p = 5.29x10^-4^; beta=-1.243; beta se=0.359) (**Fig. 2A, Sup. Tab. S1**).

We hypothesized that food patterns could be related to the observed difference in creatinine levels. From a control population that was not used in previous comparisons, individuals were selected to match the sex, age, and PC2 distribution derived from the PCA of food intake in the DLSS cohort (**Fig. 2B**). When comparing this cohort with the previously selected DLSS control cohort, we did not observe significant differences in creatinine and GFR (p=0.75; p=0.61, respectively, **Fig. 2C**).

**Table S1. Phenotypic differences between DLSS and AGE, SEX, BMI-matched controls.**

**Table S2. Principal component loadings for types of food.**

See additional xlsx file.

**References:**

1. R Core Team (2021). R: A language and environment for statistical computing. R Foundation for Statistical Computing, Vienna, Austria. URL https://www.R-project.org/
2. Weiliang Qiu (2021). powerMediation: Power/Sample Size Calculation for Mediation Analysis. R package version 0.3.4. https://CRAN.R-project.org/package=powerMediation
3. Rotar O, Moguchaia E, Boyarinova M, Kolesova E, Khromova N, Freylikhman O, et al. Seventy years after the siege of Leningrad: does early life famine still affect cardiovascular risk and aging? J Hypertens. 2015;33(9):1772-9. doi: 10.1097/HJH.0000000000000640
4. Toda C, Santoro A, Kim JD, Diano S. POMC Neurons: From Birth to Death. Annu Rev Physiol. 2017;79:209-236. doi: 10.1146/annurev-physiol-022516-034110
5. Smiley A, King D, Bidulescu A. The Association between Sleep Duration and Metabolic Syndrome: The NHANES 2013/2014. Nutrients. 2019 Oct 26;11(11):2582. doi: 10.3390/nu11112582
6. Volkov VS, Poselyugina OB, Nilova SA, Rokkina SA. Sixty years later (To 60-year anniversary since publication of G.T. Lang's book «Essential hypertension»). "Arterial’naya Gipertenziya" ("Arterial Hypertension"). 2011;17(6):555-559. (In Russ.) doi:10.18705/1607-419X-2011-17-6-555-559
